# Using Computational Approaches to Improve Risk-Stratified Patient Management: Rationale and Methods

**DOI:** 10.2196/resprot.5039

**Published:** 2015-10-26

**Authors:** Gang Luo, Bryan L Stone, Farrant Sakaguchi, Xiaoming Sheng, Maureen A Murtaugh

**Affiliations:** ^1^ School of Medicine Department of Biomedical Informatics University of Utah Salt Lake City, UT United States; ^2^ School of Medicine Department of Pediatrics University of Utah Salt Lake City, UT United States; ^3^ School of Medicine Department of Family and Preventive Medicine University of Utah Salt Lake City, UT United States; ^4^ School of Medicine Department of Internal Medicine University of Utah Salt Lake City, UT United States

**Keywords:** decision support techniques, patient care management, forecasting, computer simulation, machine learning

## Abstract

**Background:**

Chronic diseases affect 52% of Americans and consume 86% of health care costs. A small portion of patients consume most health care resources and costs. More intensive patient management strategies, such as case management, are usually more effective at improving health outcomes, but are also more expensive. To use limited resources efficiently, risk stratification is commonly used in managing patients with chronic diseases, such as asthma, chronic obstructive pulmonary disease, diabetes, and heart disease. Patients are stratified based on predicted risk with patients at higher risk given more intensive care. The current risk-stratified patient management approach has 3 limitations resulting in many patients not receiving the most appropriate care, unnecessarily increased costs, and suboptimal health outcomes. First, using predictive models for health outcomes and costs is currently the best method for forecasting individual patient’s risk. Yet, accuracy of predictive models remains poor causing many patients to be misstratified. If an existing model were used to identify candidate patients for case management, enrollment would miss more than half of those who would benefit most, but include others unlikely to benefit, wasting limited resources. Existing models have been developed under the assumption that patient characteristics primarily influence outcomes and costs, leaving physician characteristics out of the models. In reality, both characteristics have an impact. Second, existing models usually give neither an explanation why a particular patient is predicted to be at high risk nor suggestions on interventions tailored to the patient’s specific case. As a result, many high-risk patients miss some suitable interventions. Third, thresholds for risk strata are suboptimal and determined heuristically with no quality guarantee.

**Objective:**

The purpose of this study is to improve risk-stratified patient management so that more patients will receive the most appropriate care.

**Methods:**

This study will (1) combine patient, physician profile, and environmental variable features to improve prediction accuracy of individual patient health outcomes and costs; (2) develop the first algorithm to explain prediction results and suggest tailored interventions; (3) develop the first algorithm to compute optimal thresholds for risk strata; and (4) conduct simulations to estimate outcomes of risk-stratified patient management for various configurations. The proposed techniques will be demonstrated on a test case of asthma patients.

**Results:**

We are currently in the process of extracting clinical and administrative data from an integrated health care system’s enterprise data warehouse. We plan to complete this study in approximately 5 years.

**Conclusions:**

Methods developed in this study will help transform risk-stratified patient management for better clinical outcomes, higher patient satisfaction and quality of life, reduced health care use, and lower costs.

## Introduction

### Risk-Stratified Management of Chronic Disease Patients

Chronic diseases affect approximately 52% of Americans and consume 86% of health care costs [1]. Example management strategies for care include case management, disease management, supported self-care, and wellness promotion (listed in Table 1 in descending order of intensity). Each strategy is widely used and has its own benefits and properties [2,3]; for example, most major employers purchase and nearly all private health plans offer case management services [2,4] targeting early interventions at high-risk patients to prevent large expenditures and avoid deterioration of health status. Proper use of case management can reduce hospital admissions and readmissions and emergency department visits by up to 30% to 40% [3,5-9], lower costs by up to 15% [6-10], and improve patient satisfaction, quality of life, and treatment adherence by 30% to 60% [5]. A case management program can cost more than US $5000 per patient per year [6] and typically enrolls only 1% to 3% of targeted patients due to resource limitations [11]. For maximal benefit, only patients expected to incur the highest costs and/or those with the poorest prognoses should be enrolled.

**Table 1:** Description of patient management strategies.

Management strategy	Description
Case management	“A collaborative process that assesses, plans, implements, coordinates, monitors, and evaluates the options and services required to meet [a patient’s] health and human service needs” [12]. It involves a case manager who calls the patient periodically, helps make doctor appointments, and arranges for health and health-related services.
Disease management	Example intervention: check electronic medical records to find and call high-risk patients with the disease who require a specific test, but have not had it for ≥2 years.
Supported self-care	Example intervention: give patients electronic monitoring tools for self-management.
Wellness promotion	Example intervention: mail educational materials on how to maintain health.

**Figure 1:**
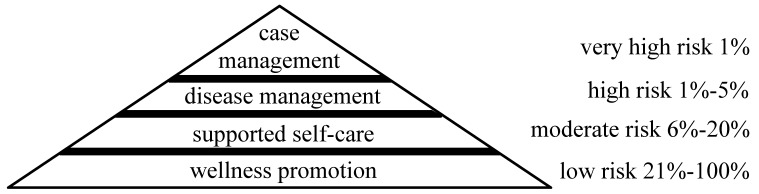
An example hierarchy of risk-stratified management levels for chronic disease patients.

Patients’ health care use and costs have a pyramid-like distribution. A small portion of patients consume most health care resources and costs [13,14]. For instance, 25% and 80% of costs are spent on 1% and 20% of patients, respectively [11,14]. High costs often result from bad health outcomes or inappropriate use of health care. Typically, more intensive management strategies are more effective at improving health outcomes, but are also more expensive. To use limited resources efficiently, risk stratification is widely used in managing patients with chronic diseases such as asthma, chronic obstructive pulmonary disease, diabetes, and heart diseases [13]. As shown in Figure 1, available management strategies are arranged into a hierarchy [14]. Patients are stratified based on predicted risk [6] and this risk can represent either high cost or a bad health outcome. Higher risk results in more intensive care to match expected returns [15]. For example, patients with predicted risk above the 99th percentile are put into case management and so on.

### Problems With the Current Risk-Stratified Patient Management Approach

The current risk-stratified patient management approach has 3 shortcomings, which result in many patients not receiving the most appropriate care and greatly degrade its outcomes.

First, existing methods for predicting individual patients’ risk have low accuracy resulting in misstratification. As shown in Allaudeen et al [16], clinicians cannot predict well which patients will become high risk in the future. Criterion-based modeling uses a priori criteria to describe high-risk patients. It is ineffective partly due to regression to the mean, in which most patients who incurred high cost or health care use in one period will stop doing so in the next period [17]. Frequently, a predictive model for individual patient health outcome or cost is used to automatically identify high-risk patients [5,18-23]. For instance, health plans in 9 of 12 communities are reported to use predictive modeling to identify candidate patients for case management [24]. For patients with predictions of the poorest outcomes or highest costs, case managers manually review patient charts and make final management decisions. Predictive modeling greatly outperforms clinicians and criterion-based modeling [17], and is the best method for identifying high-risk patients, yet needs improvement.

Existing predictive models for individual patient health outcomes and costs have low accuracy. When predicting a patient’s cost, the average error is usually as large as the average cost [25] and the *R* ^*2*^ accuracy measure is less than 20% [26]. When predicting a patient’s health outcome, the area under the receiver operating characteristic (ROC) curve accuracy measure is often low, much less than 0.8 [27-31]. These large errors cause enrollment to align poorly with patients who would benefit most from a management program [5]. As shown in Weir et al [23], among the top 10% of patients who incurred the highest costs, more than 60% were missed in the top 10% risk group selected by a predictive model. Among the top 1% of patients who incurred the highest costs, more than 80% and approximately 50% were missed in the top 1% and 10% risk groups selected, respectively. Suppose a case management program could accommodate 1% of affected patients. Even if case managers had time to manually review the top 10% risk group selected by the model and made perfect enrollment decisions, they would still miss half of the top 1% who incurred the highest costs. The case with health outcomes is similar [29,30].

Existing predictive models primarily use patient features only, implicitly assuming that a patient’s health outcome and cost depend only on the patient’s characteristics and are unrelated to the treating physician’s characteristics, which are influential. The use of treating physician’s characteristics, or physician profile features, has been exploited minimally in predictive modeling [28] leaving a knowledge gap.

Second, patients are at high risk for different reasons. Complex predictive models, including most machine learning models such as random forest, give no explanation for a prediction of high risk. Existing models also give no suggestion on interventions tailored to the patient’s specific case. An intervention addressing the reason for being at high risk tends to be more effective than nonspecific ones. For instance, for a patient who lives far from his/her physician and has difficulty accessing care, providing transportation can be effective.

A patient can be at high risk for multiple reasons each corresponding to either a single or a combination of multiple patient and physician profile features. A clinician may give the patient tailored interventions based on subjective and variable clinical judgment, but he/she is likely to miss some suitable interventions due to 3 factors:

1. Large practice variation (eg, by 1.6 to 5.6 times) exists across different clinicians, health care facilities, and regions [13,27,32-37].
2. Many features exist. A typical clinician can concurrently process no more than a single-digit number of information items [38] making it difficult to identify all these reasons due to the vast number of possible feature combinations.
3. Clinicians usually give interventions addressing patient factors only and miss those addressing physician factors. For instance, a physician may be unfamiliar with the patient’s disease. Providing the physician continuing medical education on it can be effective.

Third, thresholds for risk strata are decided heuristically with no quality guarantee leading to unnecessarily increased costs and/or suboptimal health outcomes. For instance, total future cost of all patients factoring in the management programs’ costs is unlikely to be minimized even under the unrealistic assumption that we know exactly (1) each patient’s future risk and (2) every program’s impact on each patient’s future cost if the patient is put into the program. Total future cost implicitly reflects patient health outcomes and the management programs’ benefits. For instance, fewer hospitalizations usually lead to lower costs.

### Improving Prediction Accuracy, Explaining Prediction Results, Suggesting Tailored Interventions, and Computing Optimal Thresholds

New techniques are needed to improve risk-stratified patient management so that more patients can receive the most appropriate care. To fill the gap, we will (1) combine patient, physician profile, and environmental variable features to improve prediction accuracy of individual patient health outcomes and costs; (2) develop an algorithm to explain prediction results and suggest tailored interventions; (3) develop an algorithm to compute optimal thresholds for risk strata; and (4) conduct simulations to estimate outcomes of risk-stratified patient management for various configurations. A physician’s practice profile contains his/her own information as well as clinical and administrative data of his/her patients aggregated historically. We hypothesize that using our techniques will increase prediction accuracy, improve outcomes, and reduce costs. The explanations and suggestions provided by our algorithm can help clinicians prioritize interventions and review structured attributes in patient charts more efficiently, and will be particularly useful for clinicians who are junior or unfamiliar with how to handle certain types of patients. After our methods identify patients with the highest predicted risks and give explanations and suggestions, clinicians would review patient charts, consider various factors (eg, social factors, how likely a patient’s health outcome will greatly improve [39]), and make final decisions on the management levels and interventions for these patients as is often done in case management.

### Innovation

This study is innovative for several reasons:

1. We will develop the first algorithm to (1) explain prediction results, which is critical for clinicians to trust the results and (2) suggest tailored interventions. Currently no algorithm can do the latter. Our algorithm will explain results for any predictive model without degrading accuracy and solve a long-standing open problem. In contrast, existing explanation methods are usually model specific and decrease accuracy [40,41].
2. We will transform risk-stratified patient management by personalizing management strategies based on objective data. At present, clinicians give interventions based on subjective and variable clinical judgment, and miss some of the suitable interventions for many high-risk patients.
3. The added value of physician profile features in predicting health outcomes and costs has never been systematically studied. We will include physician profile characteristics to construct new features and build new predictive models accurate for individual patients.
4. To better predict individual patient costs, we will develop a new and general technique for reducing features (ie, independent variables). The technique can increase the prediction accuracy of any continuous outcome variable with a complex nonlinear relationship with many independent variables. This is particularly useful when standard feature selection techniques [42] cannot narrow down many independent variables to a few effective features.
5. We will develop the first algorithm to compute optimal thresholds for risk strata. These thresholds aim at maximizing total expected return on the entire patient population and will be better than those determined heuristically. Currently no algorithm exists for this purpose.
6. When a predictive model is used, our study will estimate outcomes of risk-stratified patient management with multiple management strategies. No such estimates have been provided before. Previous studies have estimated outcomes for a single management strategy: case management [43].
7. We will use a new simulation method to determine which attributes are the most important to include in the predictive model. Different combinations of attributes will be used to determine the minimum performance requirement and allow tradeoffs for adapting use of our models beyond our setting based on available attributes. Previous predictive models have relied on a fixed set of attributes, which may not be collected by other sites and thus do not generalize beyond the study site.
8. Often, a specific technique is useful for only a single disease or decision support application. In contrast, after proper extension, our new techniques will generalize to a variety of decision support applications and disease settings. Examples of opportunities for future studies are (1) more precise models for health outcomes and costs will augment various decision support applications for managing limited resources, such as assisting with health care resource allocation planning [44] and automatically identifying patients likely to be admitted or readmitted in the near future triggering earlier follow-up appointments or home visits by nurses to reduce admissions and readmissions; (2) adding physician profile features can improve prediction accuracy of other outcomes, such as patient satisfaction [45], patient adherence [46], and missed appointments [47], and facilitate targeting resources, such as print and telephone reminders to reduce missed appointments [47] or interventions to improve treatment adherence [46]; (3) the algorithm for explanations and suggestions can be used to explain prediction results and suggest interventions for various applications, such as to reduce missed appointments; (4) the threshold computation algorithm can help target resources for various applications; and (5) our simulation method can be used to deploy other predictive models in clinical practice.

In summary, the significance of this study is development of new techniques to help transform risk-stratified patient management and personalize management strategies so that more patients will receive the most appropriate care. Broad use of our techniques will improve clinical outcomes, patient satisfaction, and quality of life, and reduce health care use and cost.

## Methods

Machine learning is a computer science area that studies computer algorithms improving automatically through experience. Machine learning methods, such as neural network, decision tree, and support vector machine, are widely used for predictive modeling [48] and will be used in our study. With less strict assumptions (eg, on data distribution), machine learning can achieve higher prediction accuracy, sometimes doubling it, than statistical methods [11,49,50].

### Datasets and Test Cases

This study will use a large clinical and administrative dataset in Intermountain Healthcare’s enterprise data warehouse (EDW) for all 4 aims. Intermountain Healthcare is the largest health care system in Utah, with 185 clinics and 22 hospitals. Intermountain Healthcare’s EDW contains approximately 9000 tables and an extensive set of attributes [51]. Partial lists of patient and physician attributes follow.

#### Patient Attributes

Patient attribute data include admission date and time; age; orders (eg, medications, laboratory tests, exams, immunizations, imaging, counseling), including order name, ordering provider, performing date, and result date; allergies; barriers (eg, hearing, language, learning disability, mental status, religion, vision); cause of death; chief complaint; death date; diagnoses; discharge date; exam result; facility seen for the patient visit; gender; health insurance; health care cost (eg, billed charge, Intermountain Healthcare internal cost, and reimbursed cost); height; home address; immunizations; laboratory test result; language(s) spoken; medication refills; primary care physician as listed in the electronic medical record; problem list; procedure date; procedures; provider involved in the visit; race/ethnicity; referrals; religion; visit type (eg, inpatient, outpatient, urgent care, or emergency department); vital signs; and weight.

#### Physician Attributes

Physician attribute data include age, gender, health insurances accepted, level of affiliation with Intermountain Healthcare, office location(s), specialties, type of primary care physician, and years in practice.

#### Summary Statistics of the Dataset

Our contracted Intermountain Healthcare data analyst will execute Oracle database SQL queries to extract a deidentified version of the dataset, encrypt it, and transfer it securely to a password-protected and encrypted computer on which we will perform secondary analyses. Intermountain Healthcare uses dedicated tables to track changes in diagnosis and procedure codes over time. The dataset contains information on patient encounters over the past 11 years. For the last 5 years, data captured for children cover more than 400 pediatric primary care physicians, 360,698 pediatric patients (age zero to 17 years), and 1,557,713 clinical encounters per year. Data captured for adults cover more than 600 primary care physicians, 878,448 adult patients (age ≥18 years), and 5,786,414 clinical encounters per year. Asthma prevalence is approximately 7.6% in the Intermountain Healthcare pediatric population and approximately 8.6% in the Intermountain Healthcare adult population. The dataset includes approximately 400 attributes and represents electronic documentation of approximately 85% of pediatric care and approximately 60% of adult care delivered in Utah [33,52]. Intermountain Healthcare dedicates extensive resources to data accuracy and integrity. Due to its large size and attribute richness, the dataset gives us many advantages for exploring the proposed predictive models.

In addition, we will use 21 environmental variables recorded over 11 years by regional monitoring stations within the geographic area covered by Intermountain Healthcare. These variables include particulate matter up to 2.5 μm in size (PM_2.5_) and 10 μm in size (PM_10_), carbon monoxide (CO), nitrogen dioxide (NO_2_), sulfur dioxide (SO_2_), ozone (O_3_), temperature, relative humidity, wind speed, precipitation, dew point, and activities of viruses (adenovirus; enterovirus; human metapneumovirus; influenza A virus; influenza B virus; parainfluenza virus types 1, 2, and 3; rhinovirus; and respiratory syncytial virus). Because the monitoring stations are spread across a large geographic area including the entire state of Utah, the readings of the same environmental variable can differ greatly at different monitoring stations at any time.

Using Intermountain Healthcare data, we will demonstrate our techniques on the test case of asthma patients. In the United States, asthma affects 18.7 million adults (8%) [53] and 7.1 million children (9.6%) [54,55]. Patient management strategies such as case management can ensure proper care to reduce asthma exacerbations, improve school attendance and performance, and reduce hospitalizations and emergency department visits. This impacts both quality of life and 63% of total annual asthma costs attributable to asthma exacerbations [8,56].

Our analysis results will use different combinations of attributes to determine the minimum performance requirement and allow tradeoffs for adapting use of our models beyond our setting based on available attributes. Our results will provide a cornerstone to expand testing of our techniques on other clinical datasets, patient populations, and diseases beyond asthma in the future. As patient status and feature patterns associated with high risk change over time, our techniques can be periodically reapplied (eg, to move patients across different management levels and identify newly occurring feature patterns).

##### Aim 1: Combine Patient, Physician Profile, and Environmental Variable Features to Improve Prediction Accuracy of Individual Patient Health Outcomes and Costs

###### Aim 1a: Build Predictive Models for Individual Patient Health Outcomes

###### Framework

**Figure 2:**
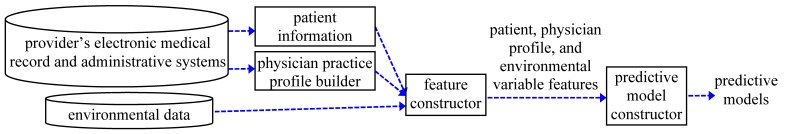
A framework for building predictive models using patient, physician profile, and environmental variable features.

We will apply the framework shown in Figure 2 to build predictive models using patient, physician profile, and environmental variable features. Environmental variables impact outcomes of certain diseases such as asthma [57,58]. The models will be used to predict individual patient health outcomes.

For each physician, we will build a practice profile including his/her own (eg, demographic) information as well as aggregated historical information of his/her patients (excluding the index patient) from the provider’s electronic medical record and administrative systems. An example physician practice profile attribute is the number of the physician’s patients with a specific disease [59]. We will use patient attributes to form patient features. We will use both patient and physician practice profile attributes to form physician profile features. Each feature is formed from one or more base attributes. If the outcome variable is affected by environmental variables, we will also use environmental variable attributes to construct features. Predictive models will be built using patient, physician profile, and environmental variable features.

There are almost an infinite number of possible features. In addition, factors such as characteristics of a pediatric patient’s parents can impact patient outcomes. This study’s purpose is not to list all possible features, exhaust all possible factors that can affect patient outcomes, and reach the theoretical limit of maximum possible prediction accuracy. Instead, our goal is to demonstrate that adding physician profile features can improve prediction accuracy and, subsequently, risk-stratified patient management. A nontrivial improvement in health outcomes and/or reduction in costs can benefit society greatly. As is typical with predictive modeling and adequate for our targeted decision support application, our study focuses on associations.

###### Data Preprocessing

We will use established techniques, such as imputation, to deal with missing values and detect and remove/correct invalid values [48,60]. For environmental variables, we will use standard methods [61,62] to obtain aggregate values, such as monthly averages, from raw values. For administrative and clinical attributes, we will use grouper models such as the Diagnostic Cost Groups system to group diseases, procedures, and drugs, and reduce features [13,25].

###### Patient Features

We will use standard patient features, such as age and diagnoses, that have been studied in the clinical predictive modeling literature [13,27,48]. Commonly used features are listed in Luo [32] and Schatz et al [29].

###### Physician Profile Features

Some physician profile features are computed using only physician practice profile attributes. Examples of such features are (1) the logarithm of the normalized number of a physician’s patients with a specific characteristic, such as a specific disease, gender, race, or age range (a logarithm is used to diminish the difference in the number across physicians); (2) the logarithm of the number of specific procedures performed by a physician; (3) the mean outcome of a physician’s patients with a specific disease (if a physician does not have enough patients with a specific disease, we will set the disease’s mean outcome in the physician’s practice profile to the mean outcome of all patients with the disease); (4) the average cost of a physician’s patients with a specific disease; (5) the average ratio of chronic controller to total asthma medications of a physician’s asthma patients, which is an asthma care quality measure [63-66]; (6) the mean of a feature of a physician’s (pediatric) asthma patients with desirable/undesirable outcomes; (7) a physician’s age; (8) a physician’s total office hours per week; (9) a physician’s years in practice; and (10) a physician’s specialty.

Some physician profile features are formed by combining patient and physician practice profile attributes, characterizing the match of patient and physician. Examples of such features are (1) the distance between the physician’s office and patient’s home, (2) an indicator of whether the physician and patient are of the same gender [67], (3) an indicator of whether the physician and patient speak the same language, and (4) an indicator of whether the physician accepts the patient’s insurance.

The preceding lists of physician profile features are only for illustration purposes and are by no means exhaustive. More physician profile features will be investigated in this study. When a patient is managed by multiple physicians simultaneously, the patient’s outcomes are affected by the profile features of all these physicians. A traditional method for handling this situation is to use episode grouper software to split the whole span of patient care into episodes and assign each episode to a single physician [13,68]. An episode of care is “a series of temporally contiguous health care services related to treatment of a given spell of illness or provided in response to a specific request by the patient or other relevant entity” [27,69]. Apart from the episode method, we will investigate other methods to combine multiple physicians’ profile features.

###### Environmental Variable Features

We will use standard environmental variable features such as monthly averages from clinical predictive modeling literature [57].

###### Definition of Asthma Cases and Outcomes

As test cases, we will focus on primary care physicians and develop and test our idea using (1) pediatric asthma and (2) adult asthma. The method described in Schatz et al [29,70,71] will be used to identify asthma patients. A patient is considered to have asthma if he/she has (1) at least one *International Classification of Diseases, Ninth Revision* (*ICD-9*) diagnosis code of asthma (493.xx) or (2) at least 2 asthma-related medication dispensing records (excluding oral steroids) in a 1-year period, including inhaled steroids, beta-agonists (excluding oral terbutaline), oral leukotriene modifiers, and other inhaled antiinflammatory drugs [29]. We will use 2 outcome measures for asthma: (1) primary outcome—whether acute care (inpatient stay, urgent care, and emergency department visit) with a primary diagnosis of asthma (*ICD-9* code: 493.xx) occurred for a patient in the following year [28,29,31,32,56,72,73] and (2) secondary outcome—the total amount of reliever medication and oral steroid medication for acute asthma exacerbations that a patient refilled in the following year. Total refill amount reflects the number and degree of asthma exacerbations experienced by the patient [63,64] and is available in our dataset.

###### Predictive Models

We will use Weka [74], a widely used open-source machine learning and data mining toolkit, to build predictive models. Weka integrates an extensive set of popular machine learning algorithms, ensemble techniques combining multiple predictive models, feature selection techniques, and methods for handling the imbalanced class problem. Both numerical and categorical variables appear in clinical, administrative, and environmental data. We will use supervised algorithms that can handle both types of variables, such as decision tree and *k*-Nearest Neighbor. We will test every applicable algorithm and manually tune hyperparameters.

The accuracy achieved by state-of-the-art predictive models is usually far below 80% [28,29]. We would regard Aim 1a (to build predictive models for individual patient health outcomes) partially successful if we can improve accuracy by 10% or more for either pediatric or adult asthma. We would regard Aim 1a completely successful if we can improve accuracy by 10% or more for both pediatric and adult asthma. Given a set of features, we will use 3 methods to improve model accuracy. First, some features are unimportant or highly correlated with one another, which may degrade model accuracy. To address this, we will use standard feature selection techniques, such as the information gain method, to identify important features that will be used in the model [28,42,74]. Second, for a categorical outcome variable with 2 values, the corresponding 2 classes in our dataset can be imbalanced, meaning many more instances exist for one class than the other. This can potentially degrade model accuracy. We will use standard techniques such as Synthetic Minority Oversampling Technique (SMOTE) to address this [74]. Third, we will try ensemble techniques, such as random forest, that combine multiple models and usually work better than individual models [74].

###### Accuracy Evaluation and Sample Size Justification

We have 11 years’ data. We will use a standard approach to train and test predictive models. We will conduct stratified 10-fold cross validation [74] on the first 10 years’ data to train and estimate the accuracy of models. The 11th year’s data will be used to assess the best models’ performance reflecting use in practice. For categorical outcome variables, we will use the standard performance metric of the area under the curve (AUC) of the ROC [74] to select the best model. For continuous outcome variables, we will use the standard performance metric of *R* ^*2*^ to select the best model and also report the Cumming’s prediction measure (equivalent to the mean absolute prediction error) [25,32]. To determine the clinical, administrative, and environmental variable attributes essential for high accuracy, backward elimination [48] will be used to drop independent variables as long as the accuracy does not drop by more than 0.02.

We will test the hypothesis that adding physician profile features can increase prediction accuracy twice—once for children and once for adults. We will compare the accuracies achieved by 2 predictive models using the best machine learning algorithm. The first model will use patient, physician profile, and environmental variable features; the second, only patient and environmental variable features. We will accept the hypothesis if the first model achieves higher accuracy (AUC or *R* ^*2*^) than the second model by 10% or more.

Consider the categorical outcome variable of acute care usage with 2 values (classes). A predictive model using only patient and environmental variable features usually achieves an AUC far less than 0.8 [28,29]. Using a 2-sided *z* test at a significance level of .05 and assuming for both classes a correlation coefficient of .6 between the 2 models’ prediction results, a sample size of 137 instances per class has 90% power to detect a difference of 0.1 in AUC between the 2 models. The 11th year’s data include approximately 27,000 children and 75,000 adults with asthma, providing adequate power for testing our hypothesis. To train a predictive model well, typically the ratio of the number of data instances to the number of features should be 10 or more. In our case, a few hundred features at most will be used; thus, our dataset would be large enough for training the predictive models. The case with the continuous outcome variable is similar (see Aim 1b: Sample Size Justification).

#### Aim 1b: Build Predictive Models for Individual Patient Costs.

We will use an approach similar to that in Aim 1a, but change the prediction target from health outcomes to individual patients’ total costs in the following year [13,25,27]. Each medical claim is associated with a billed cost, an Intermountain Healthcare internal cost, and a reimbursed cost [13]. We will use the Intermountain Healthcare internal cost [33], which is less subject to variation due to member cost-sharing [13], and reflects actual cost more closely. To address inflation, we will standardize all costs to 2014 US dollars using the medical consumer price index [75].

In addition to the rare use of physician profile features, 2 other major reasons also cause low accuracy in predicting an individual’s cost. First, most existing work on predicting costs uses linear regression models [13,25,27]. In reality, costs do not follow a linear model [26]. Second, the cost of a patient with a specific disease is the cost of treating all his/her diseases [25]. To consider this factor, each model uses many features or independent variables (eg, one feature per disease) and can easily have insufficient training data [48]. To address these 2 problems, we will try nonlinear, disease-specific, machine learning models, which were proposed in a previous paper [32], but have not been implemented so far. This method’s key idea is to reduce features by merging several less important features into one feature while maintaining important features as separate. The current approach of identifying important features and grouping other features is manual. We will also investigate automatic approaches. For example, we can regard the top features with the largest associations with the outcome variable as important ones. The remaining features are clustered using a similarity metric to form groups. The automatic approach is general and can be used to improve prediction accuracy of any continuous outcome variable that has a complex nonlinear relationship with many independent variables.

##### Sample Size Justification

In predicting an individual’s cost, a predictive model using only patient and environmental variable features usually achieves an *R* ^*2*^ <20% [26]. Using an *F* test at a significance level of .05 and assuming the presence of 70 patient and environmental variable features, a sample size of 245 patients has 90% power to detect an increase of 10% in *R* ^*2*^ attributed to 30 physician profile features. The 11th year’s data include approximately 27,000 children and 75,000 adults with asthma, providing adequate power for testing our hypothesis of an increase of 10% or more in *R* ^*2*^.

Our goal is to achieve a 10% or more improvement in accuracy. If our models cannot achieve high accuracy on the entire group of asthma patients, we will build separate models for different subgroups of asthma patients. Patient subgroups are defined by specific characteristics, such as age, prematurity, comorbidity, or insurance type that are usually independent variables of the original models. If our models still cannot achieve high accuracy, we will conduct subanalyses to identify patient subgroups on which our models perform well. In this case, our final models will be applied only to the identified patient subgroups.

A missing data problem occurs when a patient has several physicians belonging to different provider groups, with no single provider having complete information on the patient. We anticipate that adding physician profile features can improve prediction accuracy even if some data are missing. The missing data problem is unlikely to be an issue for children in our case, as Intermountain Healthcare provides approximately 85% of pediatric care in Utah [52]. If the Intermountain Healthcare EDW misses too much data for adults, we will use claim data in the all-payer claims database [76] to compensate. In the future when applying our predictive models to other health care systems, this compensation strategy can be used. Also, we expect missing data problems to be uncommon in health maintenance organization settings where all physicians managing the patient belong to the same provider group and the provider’s electronic medical record and administrative systems usually have all medical data collected on the patient [77].

As mentioned previously, identifying asthma requires medication order and refill information. Our dataset includes this information because Intermountain Healthcare has its own health insurance plan (SelectHealth [78]). If the Intermountain Healthcare EDW is missing too much refill information, we will use claim data in the all-payer claims database [76] to compensate. If adding physician profile features cannot significantly increase prediction accuracy for asthma, we will choose chronic obstructive pulmonary disease or heart diseases for Aims 1 to 4.

We have a large dataset. If we experience scalability issues using Weka, we will use a parallel machine learning toolkit, such as Spark’s MLlib [79-81], to build predictive models on a secure computer cluster available to us at the University of Utah Center for High Performance Computing [82].

### Aim 2: Develop an Algorithm to Explain Prediction Results and Suggest Tailored Interventions

For patients with predicted risk greater than a predetermined threshold, such as the 95th percentile, this aim will explain prediction results and suggest tailored interventions. These explanations and suggestions can help clinicians make final decisions on the management levels and interventions for these patients.

Prediction accuracy and model interpretability are frequently 2 conflicting goals. A model achieving high accuracy is usually complex and difficult to interpret. How to achieve both goals simultaneously has been a long-standing open problem. Our key idea to solve this problem is to separate prediction and explanation by using 2 models concurrently, each for a different purpose. The first model makes predictions and targets maximizing accuracy. In this study, this model is the best one built for the outcome variable in Aim 1. The second model is rule-based and easy to interpret. It is used to explain the first model’s results rather than make predictions. The rules used in the second model are mined directly from historical data rather than coming from the first model. For each patient whom the first model predicts to be at high risk, the second model will show zero or more rules. Each rule gives a reason why the patient is predicted to be at high risk. Because some patients can be at high risk for rare reasons that are difficult to identify, we make no attempt to ensure that at least one rule will be shown for every patient predicted to be at high risk. Instead, we focus on common reasons that are more important and relevant to the patient population than rare ones. We expect most high-risk patients to be covered by one or more common reasons.

We will use an associative classifier [83-85] from the data mining field as the second model. Associative classifiers can handle both numerical and categorical variables and be built efficiently from historical data. Compared with several other rule-based models, an associative classifier includes a more complete set of interesting and useful rules and can better explain prediction results. For ease of description, our presentation focuses on the case that each patient has exactly one data instance (row). The case in which a patient has more than one data instance can be handled similarly. We will proceed in 3 steps.

In step 1, association rules are mined from historical data. As mentioned in Aim 1, each patient is described by the same set of patient, physician profile, and environmental variable features and labeled as either high risk or not. An associative classifier includes a set of class-based association rules. Each rule includes a feature pattern associated with high risk and is of the form: *p*_*1*_ AND *p*_*2*_ AND ... AND *p*_*k*_ is associated with high risk. The value of *k* varies across different rules. Each item *p*_*i*_ (1≤*i*≤*k*) is a feature-value pair of the form (*f*, *v*) indicating that feature *f* takes a value equal to *v* (if *v* is a value) or within *v* (if *v* is a range). The rule suggests that a patient is likely to be at high risk if he/she satisfies *p*_*1*_, *p*_*2*_, ... , and *p*_*k*_. An example rule is the patient was hospitalized for asthma last year AND the patient’s primary care physician has fewer than 10 asthma patients is associated with high risk.

For a given association rule, the percentage of patients satisfying the rule’s left side and being at high risk reflects the rule’s coverage and is called the rule’s *support*. Among all patients satisfying the rule’s left side, the percentage of patients at high risk reflects the rule’s accuracy and is called the rule’s *confidence*. An associative classifier includes association rules at a given level of minimum support (eg, 1%) and confidence (eg, 70%). These rules can be efficiently mined from historical data using existing techniques [83-85], which can eliminate redundant and noisy rules. Because we need only rules suggesting high risk, we can mine desired feature patterns (ie, the rules’ left side) from high-risk patients’ data rather than from all patients’ data to improve the efficiency of rule generation.

Typically, many association rules will be mined from historical data [83-86]. Keeping all these rules will overwhelm clinicians. To address this issue, we will use 3 methods to reduce the number of rules. First, in forming rules, we will consider only features appearing in the first model that is used to make predictions. As mentioned in Aim 1a, many nonessential features will be removed during feature selection and backward elimination when building the first model. Second, we will focus on rules with no more than a predetermined small number of items (eg, 4) because long feature patterns are difficult to understand and act on [83]. Third, users can optionally specify for a feature what values or type of range (eg, stating that the feature is above a threshold) may potentially indicate high risk and appear in rules [40,87]. The other values or types of range are not allowed to appear in rules. This also helps form clinically meaningful rules.

In step 2, interventions will be listed for the mined association rules. Through discussion and consensus, our clinical team will examine mined association rules and remove those that make little or no clinical sense. For each remaining rule, the clinicians will list zero or more interventions addressing the reason given by the rule. Example interventions for patients include (1) provide transportation or telemedicine for a patient living far from his/her physician, (2) schedule longer or more frequent doctor appointments for a patient with multiple comorbidities, (3) schedule appointments with nurse educators or clinical pharmacists for a patient with multiple comorbidities, (4) arrange language service for a doctor appointment if the patient and physician speak different languages, and (5) give wearable air purifiers to certain types of asthma patients living in an area with bad air quality.

Example interventions at the system level include (1) provide the primary care physician continuing medical education on a specific disease, cultural competence, women’s health, or pediatric health if he/she is unfamiliar with or cannot well manage the disease, patients of a particular race, diseases in women, or pediatric diseases (a physician may be unfamiliar with a disease if he/she has few patients with it; a bad mean outcome of a physician’s patients with the disease may indicate, but not always, that the physician cannot manage the disease well); (2) extend physician office hours; and (3) open a new primary care clinic in an area with no such clinic nearby.

Interventions for patients are displayed to clinicians in step 3. Interventions at the system level are optional and may be viewed only by managers of the health care system. We call a rule actionable or nonactionable based on whether or not at least one intervention is associated with it. The remaining rules and their associated interventions will be stored in a database to facilitate reuse.

In step 3, prediction results are explained and tailored interventions are suggested. At prediction time, for each patient identified as high risk by the first model, we will find all association rules whose left side is satisfied by the patient using an index for rules [84]. We will display the actionable rules above the nonactionable ones, each in descending order of confidence [84]. If 2 rules have equal confidence, the rule with higher support will be ranked higher. If 2 rules have the same confidence and support, the one with fewer items will be ranked higher. Our rule sorting method differs from several traditional ones [83-85] because our goal is to explain the prediction result for a patient rather than to maximize the average prediction accuracy in a patient group. We will list confidence and associated interventions, if any, next to each rule to help the clinician identify suitable tailored interventions. By default, we will show no more than a predetermined small number of rules (eg, 3). If desired, the clinician can opt to view all rules applicable to the patient.

Commonly used support and confidence thresholds [83-85] may not be suitable for our case, in which only a small percentage of patients are at high risk. We will adjust the support and confidence thresholds if the commonly used ones cannot produce enough meaningful association rules. By setting the thresholds low enough, we will produce meaningful rules at the expense of our clinicians spending time removing rules that make little or no clinical sense. Because existing predictive models give no suggestion on tailored interventions, we will regard Aim 2 successful if a nontrivial percentage (eg, ≥20%) of high-risk patients are covered by actionable rules.

#### Performance Evaluation

The algorithm for explanations and suggestions will be evaluated in Aim 4.

### Aim 3: Develop an Algorithm to Compute Optimal Thresholds for Risk Strata

In risk-stratified management, chronic disease patients are stratified into multiple levels [14,15]. This aim will compute the optimal thresholds for these levels that minimize total future cost of all patients factoring in the management programs’ costs. Total future cost implicitly reflects patient health outcomes, health care use, efficiency of care, and the management programs’ benefits. For instance, fewer hospitalizations usually lead to lower costs. The following discussion focuses on stratification based on predicted patient risk of experiencing a specific type of undesirable event (eg, hospitalization or emergency department visit). The case of stratification based on predicted cost or with more than one type of undesirable event can be handled similarly. Our discussion applies to any predictive model and is based on a fixed period in the future, such as the next 12 months.

#### Threshold Computation Algorithm

We will conduct quantitative analysis to determine the optimal management level for each risk percentile. We will proceed through the risk percentiles one by one, from the highest to the lowest. Given a risk percentile, we will compute for each management level the average future cost per patient in the percentile if patients in the percentile are put into the level. The level with capacity remaining in its management program and the lowest average future cost per patient will be chosen for the risk percentile.

More specifically, consider a risk percentile and an average patient whose predicted risk falls into the percentile. If the patient is enrolled in a management program, we estimate that the patient’s future cost will change by delta = the program’s cost – the program’s benefit gained by reducing undesirable events=*c*_*i*_–*avg_n*_e_*p*c_*e*_ compared with no enrollment. Here, *c*_*i*_ is the program’s average cost per patient. Factors such as increased medication cost due to better medication adherence are included in *c*_*i*_. *avg_n*_*e*_ is the average number of undesirable events that a patient in the risk percentile will experience in the future. *p* is the percentage of undesirable events the management program can help avoid, reflecting the program’s benefit. *c*_*e*_ is the average cost of experiencing the undesirable event once. *c*_*i*_ and *p* can be obtained from statistics reported in the literature for the management program [39,88]. *avg_n*_*e*_ can be obtained by making predictions on historical data and checking the corresponding statistics for the risk percentile. *c*_*e*_ is obtained from statistics on historical data. The management level with the smallest delta is optimal for the risk percentile. If no statistics on *c*_*i*_ and *p* of a management program are available in the literature, the clinician in our research team (Dr Stone) will provide rough estimates based on experience. We will perform sensitivity analysis when choosing thresholds by varying the estimated values of *c*_*i*_ and *p* to obtain the full spectrum of possible outcomes in Aim 4.

The preceding method performs an exhaustive search among all management levels for each risk percentile. In practice, we would expect *avg_n*_*e*_ to decrease as the predicted patient risk of experiencing undesirable events becomes smaller. We will investigate using this property to reduce the search space when going through the risk percentiles one by one, from the highest to the lowest.

#### Performance Evaluation

The threshold computation algorithm will be evaluated in Aim 4.

### Aim 4: Conduct Simulations to Estimate Outcomes of Risk-Stratified Patient Management for Various Configurations

To determine a predictive model’s value for future deployment in clinical practice, we need to estimate outcomes of risk-stratified patient management when the model is used and determine how to generalize the model to differing sites collecting different sets of attributes. Our models will be built on Intermountain Healthcare datasets. Our simulations will guide how to deploy the models in another health care system. No previous study has either estimated outcomes for a model with more than one management strategy or determined the attributes most important for generalizing the model. We will demonstrate our simulation method for the task of risk-stratified management of (1) asthmatic children and (2) asthmatic adults by using our models for predicting acute care use for asthma in the following year (see Aim 1a: Definition of Asthma Cases and Outcomes), the hierarchy of risk-stratified management levels shown in Figure 1, and our algorithms described in Aims 2 and 3. Our simulation method is general and can be used to deploy other models in clinical practice. We will first evaluate the technique in Aim 1.

#### Outcomes

We will focus on the outcomes of costs, hospital admissions, and emergency department visits in the following year. Cost is the primary outcome, reflecting health care use and efficiency of care. Other outcomes are secondary and are indirectly reflected in costs.

#### Estimate Outcomes

Given a set of attributes and a predictive model, we will estimate each outcome. We will use the same method as in Aim 1 to train the model on the first 10 years’ data. For the 11th year’s data, we will obtain prediction results, compute thresholds for risk strata, then estimate the outcome in a way similar to Aim 3. For example, consider a patient who will have a cost of *h* and experience *n*_*e*_ undesirable events in the following year with no program enrollment. If the patient is enrolled in a management program, we estimate that the patient’s future cost will become *h* + *c*_*i*_–*n*_e_*p*c_*e*_, where *c*_*i*_, *p*, and *c*_*e*_ are as defined in Aim 3. The overall outcome estimate is the aggregate of estimated outcomes for all patients. Using a similar approach, we can identify the minimum accuracy requirement of the model for it to be clinically valuable.

#### Sensitivity Analysis

Intermountain Healthcare collects an extensive set of attributes. Another health care system may collect only a subset of these attributes. To ensure the model’s generalizability, we will test various combinations of attributes and estimate outcomes when the modified model is used. The estimate will identify which attributes are critical. If an important attribute is unavailable in a specific health care system, the estimate can suggest alternative attributes with minimal negative impact on outcomes.

Our full model will use up to 400 attributes. It is not possible to conduct simulations for every possible combination of these attributes. Instead, we will use an attribute grouping approach associating attributes likely to coexist, such as attributes associated in a laboratory test panel, based on our clinical expert’s judgment. We will construct and publish a table listing possible combinations of attributes by groups, including outcomes estimated through simulations and the predictive model’s trained parameters. A health care system interested in deploying the model can use the table to determine expected outcomes for their data environment and identify attributes that need to be collected. One entry in the table will correspond to the attributes available in the Observational Medical Outcomes Partnership (OMOP) common data model [89], which standardizes clinical and administrative attributes from more than 10 large health care systems in the United States [90]. The model in this entry will directly apply to at least those health care systems. If conducting simulations for the many combinations of attribute groups is too slow on one computer, we will parallelize simulations on a secure computer cluster available to us [82].

#### Outcome Evaluation and Sample Size Justification

We will compare outcomes achieved by 2 predictive models using the best machine learning algorithm. The first model will use patient, physician profile, and environmental variable features; the second only patient and environmental variable features. We will test 3 hypotheses: adding physician profile features will be associated with reduced (1) costs, (2) hospital admissions, and (3) emergency department visits. We will test each hypothesis twice, once for children and once for adults. Cost data will be log-transformed due to skewed distribution [13]. We will accept the primary hypothesis if the first model can reduce the log cost by 10% multiplied by its standard deviation compared with the second model. One-sided paired-sample *t* test will be used to test the difference in log cost between the 2 models’ outcomes. McNemar’s test will be used to test the difference in hospital admissions and emergency department visits. At a significance level of .05, a sample size of 857 instances has 90% power to confirm the primary hypothesis. The 11th year’s data include approximately 27,000 children and 75,000 adults with asthma, providing adequate power for testing the primary hypothesis.

We will do 2 similar analyses to compare our threshold computation algorithm versus the current method of determining thresholds heuristically (evaluate the technique in Aim 3) and our algorithm for explanations and suggestions versus the current method of giving no explanation and suggestion (evaluate the technique in Aim 2). Physician profile features will be used in either analysis. In the first analysis, we will use the heuristically determined thresholds reported in the literature [15]. In the second analysis, we will use our threshold computation algorithm and estimate outcomes of our algorithm for explanations and suggestions. For an intervention, we will use statistics on its benefits and average cost per patient from the literature [39] where available. If no information is available, the clinician in our research team (Dr Stone) will conservatively estimate these numbers’ minimum and maximum values based on experience. For each number, we will use 5 levels ranging from the minimum to the maximum value. To obtain the entire spectrum of possible outcomes, we will perform sensitivity analysis by varying the level and percentage of suggested interventions that clinicians will use. For the current method of giving no explanation and suggestion, we will proceed in a similar way by letting Dr Stone estimate the lower and upper bounds of the likelihood that clinicians will use an intervention. If Dr Stone has difficulty estimating the likelihood that clinicians will use an intervention, we will interview clinicians using sample patient cases to help with the estimation. Based on its own estimate of the situation, a health care system can check where in the spectrum it will fall.

### Ethics Approval

We have already obtained institutional review board approvals from the University of Utah and Intermountain Healthcare for this study.

## Results

We are currently in the process of extracting clinical and administrative data from the Intermountain Healthcare EDW. We plan to complete this study in approximately 5 years.

## Discussion

Our techniques’ principles are general and rely on no special property of any disease, patient population, or health care system. Just as predictive models are used for case management for various diseases and patient populations [13,24,30,31], after proper extension our techniques can be used for a range of decision support applications in various settings (see the innovation subsection of the Introduction). Our simulation method will determine how to generalize a predictive model to differing sites collecting different sets of attributes and the attributes most important for generalization. Using data from an integrated health care system with many heterogeneous facilities spread over a large geographic area, we will demonstrate our techniques on the test case of asthma patients. These facilities include 22 hospitals and 185 clinics, ranging from tertiary care hospitals in metropolitan areas staffed by subspecialists to community urban and rural clinics staffed by family physicians and general practitioners with limited resources. Variation in geographic location, patient population, cultural background, staff composition, and scope of services provides a realistic situation to identify factors generalizable to other facilities nationwide. When conducting simulations for each disease (pediatric/adult asthma), one of the models produced will directly apply to 10 or more large health care systems.

Because inaccurate predictive models are commonly used already for case management [24], we would expect our more precise models to have practical value. Future studies will demonstrate our techniques on other diseases, test cases, and patient populations, implement our techniques in a major health care system for risk-stratified management of asthmatic children, and test the impact in a randomized controlled trial.

In summary, our work will transform risk-stratified patient management and personalize management strategies based on objective data so that more patients will receive the most appropriate care. This will improve clinical outcomes and reduce health care use and cost. We will achieve generalizable advances in predictive modeling, explaining prediction results, tailoring interventions, and resource allocation. After proper extension, our new techniques can be used for a variety of decision support applications in various disease settings. The new simulation method will be useful for estimating outcomes for a predictive model in dissimilar data environments.

## Abbreviations

AUC: area under the curve
EDW: enterprise data warehouse
ICD-9: International Classification of Diseases, Ninth Revision
OMOP: Observational Medical Outcomes Partnership
ROC: receiver operating characteristic
SMOTE: Synthetic Minority Oversampling Technique
